# Network Structure and Properties of Lithium Aluminosilicate Glass

**DOI:** 10.3390/ma15134555

**Published:** 2022-06-28

**Authors:** Shoujia Huang, Wenzhi Wang, Hong Jiang, Huifeng Zhao, Yanping Ma

**Affiliations:** 1 State Key Laboratory of Marine Resources Utilization in South China Sea and Special Glass Key Lab of Hainan Province, Hainan University, Haikou 570228, China; hsjia1643853146@163.com (S.H.); 20085600210057@hainanu.edu.cn (W.W.); myp@hainanu.edu.cn (Y.M.); 2 Chengmai and State Key Laboratory of Special Glass, HNHT Special Glass Technology Co., Ltd., Chengmai 571924, China; zhaohuifeng19673@126.com

**Keywords:** lithium aluminosilicate glass, network structure, boron oxide, chemical stability, mechanical properties

## Abstract

Based on lithium aluminosilicate glass, the composition of glass was optimized by replacing SiO_2_ with B_2_O_3_, and the influence of glass composition on structure and performance was studied. With the increase in B_2_O_3_ concentrations from 0 to 6.5 mol%, Al_2_O_3_ always existed in the form of four-coordinated [AlO_4_] in the network structure, and B_2_O_3_ mainly entered the network in the form of four-coordinated [BO_4_]. The content of Si-O-Si linkages (Q_4_^(0Al)^) was always dominant. The incorporation of boron oxide improved the overall degree of polymerization and connectivity of the lithium aluminosilicate glass network structure. An increase in the degree of network polymerization led to a decrease in the thermal expansion coefficient of the glass and an increase in Vickers hardness and density. The durability of the glass in hydrofluoric acid and NaOH and KOH solutions was enhanced overall.

## 1. Introduction

B_2_O_3_ has been incorporated into various glass systems to obtain the required chemical and physical properties [1]. Boron-containing silicate glass is a commonly used material in industrial applications and has been widely used in applications in industrial technology fields including pharmaceutical containers, sealing glass, optics, flat-panel displays, electronic packaging and low-expansion glass [2,3,4,5]. The ability of the modifiers and network formers in the glass components to compete for oxygen affects the coordination of boron, that is, the glass component plays an important role in the coordination of boron. Therefore, the structure of boro-aluminosilicate glass is more complicated [6]. In pure boron oxide glass, boron is three-coordinated. With the addition of alkali metal oxides, alkali metal ions compensate for the charge of the [BO4]- unit, and four-coordinated boron begins to appear [7]. Borate or borosilicate glass exhibits nonlinear composition–property relationships, which is a phenomenon known as the “boron anomaly” [8,9,10]. The relationship between the composition, performance and structure of traditional borosilicate glass has been extensively and systematically studied [11,12,13,14,15,16]. For instance, Yun and Bray [17] put forward a model for calculating fraction N4 of tetra-coordinated boron. According to the model, when the ratio of sodium oxide to boron oxide exceeds 0.5, there are enough sodium ions to compensate the negative charge of [BO4] units, and the number of [BO4] units in glass increases; on the contrary, the number of [BO3] units is greater. Jonathan F. Stebbins [18] quantified the number of non-bridging oxygens bonded to boron in sodium borate glass and barium borate glass, which have a major influence on glass properties, and enlightened the quantification of the number of non-bridging oxygens in borate networks in multicomponent glass. Du and Stebbins [19] improved the model established by “Dell, Bray and Xiao [20]”. The improved model can predict the non-bridging oxygen (NBO) content and N_4_ only based on the composition of the glass. The experimental data on aluminoborosilicate glass are highly consistent.

Compared with traditional borosilicate glass, for which a relatively well-established structural model has been established, the structure of lithium aluminosilicate glass containing various alkali metal and alkaline earth metal oxides is more complex. The high-field cations introduced by alkaline earth metal oxides affect the form of aluminum and boron atoms in the network structure, resulting in their variable roles in the network structure. Abd El-Moneim et al. [21] found that in ternary alkaline earth aluminoborate glass RO-Al_2_O_3_-B_2_O_3_, where R = Mg, Ca and Sr, the network connectivity of magnesium-doped aluminoborate glass was the strongest, and the effect of Ca on enhancing network connectivity was stronger than that of Sr. In alkali-free aluminoborosilicate glass, with the increase in alkaline earth metal oxides, aluminum always exists in the four-coordinated form. Boron is mainly three-coordinated; four-coordinated boron increases the elastic modulus and elasticity of the glass, and the density increases as it increases, while the acid resistance decreases [22].

The structure of glass has an important influence on its properties. Raman spectroscopy and nuclear magnetic resonance spectroscopy are two complementary methods for studying the glass structure. Manara et al. [23] summarized the vibration types of boron using Raman spectroscopy, and the borate vibrations were concentrated in the low-frequency region of <1000 cm^−1^. In the region of 800–1200 cm^−1^, there were silicon–oxygen tetrahedrons with different bridging-oxygen number (Q^n^, where n is the bridging oxygen number, with 0 ≤ n ≤ 4) stretching vibrations [24,25,26,27,28,29,30]. The vibration of part of the boron species in this region overlaps with the vibration of the silicon–oxygen tetrahedron [31,32,33,34,35,36,37]. Therefore, it is difficult to quantitatively analyze the boron species by Raman spectroscopy alone. NMR has been widely used in glass structures. ^27^Al MAS NMR provides information about the amount of four-coordinated aluminum and can identify the amount of Al^IV^ incorporated in the network; the ^29^Si MAS NMR data also provide information about the glass network structure and the number of Si-O-Al linkages connected by bridging oxygen; the broad spectrum of ^11^B MAS NMR is used to determine the content of [BO_4_] and [BO_3_] groups, including symmetric and asymmetric triangular boron groups [38,39].

In this paper, a high-temperature melting method was used to prepare a lithium aluminum silicate (LAS) glass sample with a molar composition of 3.75Na_2_O-5.94MgO-10.06Al_2_O_3_-(71.64-x) SiO_2_-8.62Li_2_O-xB_2_O_3_. The effects of B_2_O_3_ on the network structure and density, molar volume, chemical stability, and mechanical properties of glass were studied and analyzed by means of nuclear magnetic resonance spectroscopy and Raman spectroscopy, and the relationship among glass composition, structure and performance was analyzed.

## 2. Materials and Methods

### 2.1. Glass Preparation and Characterization

The main raw materials of LAS glass prepared using the high-temperature melting method were: ultra-white silica sand (SiO_2_; 99.7%), sodium carbonate (Na_2_CO_3_; 99%), magnesium oxide (MgO; 99.5%), lithium carbonate (Li_2_CO_3_; 99%), aluminum hydroxide (Al(OH)_3_; 99.47%) and boric acid (H_3_BO_3_; 99.92%), using NaCl as clarifying agent. The molar composition of boron-doped LAS glass was: 3.75Na_2_O-5.94MgO-10.06Al_2_O_3_-(71.64-x)SiO_2_-8.62Li_2_O-xB_2_O_3_(x = 0,1.5,2.5,3.5,4.5,5.5,6.5). We put the weighed raw materials into a quartz crucible, which we heated in a high-temperature furnace at 1600 °C for 2 h; then, we took out the molten glass and poured it into a stainless-steel mold for casting; then, we put it in an annealing furnace at 600 °C for 2 h, followed by cooling down to room temperature within the furnace.

It was likely that a significant proportion of boron volatilized at high temperatures and caused deviations from nominal compositions. With this in mind, an additional 12% B_2_O_3_ was added to each sample to compensate for volatilization prior to melting. For example, 1.5 mol% B_2_O_3_ for 2# was equivalent to 1.72 wt% oxide, and we added an additional 1.72 × 12% g B_2_O_3_ per 100 g of oxide in a well-mixed batch. The practical proportion and volatility of B_2_O_3_ measured by chemical analysis are shown in Table 1. The test method was in accordance with the Chinese national standard method. From the quantitative analysis results of ICP and XRF, the volatiles of B_2_O_3_ in the samples were not exactly the same, but the actual concentration of B_2_O_3_ was close to the nominal value. As shown in Table 1, the deviation of boron content was not more than 5%. Deviations from nominal components were, therefore, ignored in the calculations to simplify the understanding process.

### 2.2. Glass Testing and Characterization

Solid-state ^29^Si NMR spectra were obtained using a Bruker AVANCE III HD 400 MHz solid-state NMR spectrometer, which had an H/X dual resonance solid probe, a 4 mm ZrO_2_ rotor and a rotor speed of 14 kHz; the detection resonance frequency of the ^29^Si NMR spectrum is 79.49 MHz, and the magnetic field strength is 9.4 T, while scan times are generally 512 times an hour. ^27^Al NMR spectra were obtained using a Bruker AVANCE III HD 400 MHz solid-state NMR spectrometer, which also had an H/X dual-resonance solid-state probe, a 4 mm ZrO_2_ rotor, and a rotating speed of 14 kHz. The detection resonance frequency of ^27^Al is 104.26 MHz, and the number of scans is generally 4096 times an hour. ^11^B solid-state NMR spectra were acquired using a Bruker AVANCE NEO 400 WB solid-state superconducting NMR spectrometer (Fällanden, Switzerland), with samples loaded in 4 mm ZrO_2_ sample tubes with a Bruker 4 mm standard probe, using the single-pulse technique. The resonance frequency of the boron spectrum is 128.39 MHz. The deconvolution of the NMR spectra was performed with DM-fit software [40]. When only the central part of the spectrum was considered, the non-linear least squares method was used to iteratively determine the positions of different components and calculate the corresponding areas. The accuracy of the chemical shift value of the NMR spectrum was estimated to be 0.2 ppm, and the accuracy of the area quantification was estimated to be 3%. Raman excitation was applied using a solid-state 532 nm wavelength laser and an output power set to 40 milliwatts. The acquisition time for a given sample was usually between 20 and 60 s, and 6 scans were accumulated. In order to determine whether the glass samples were uniform, at least 4 spectra were collected for each sample. Subtract the background using a third-order polynomial function anchored in the unbanded region: ~200 cm^−1^, 850 cm^−1^, 1200 cm^−1^, and 1600 cm^−1^. Raman spectra were acquired at room temperature without correction for the temperature and frequency dependence of scattering intensity. At room temperature, a third-order polynomial function anchored in the non-band region was used to subtract the spectrum from the spectrum background from 200 to 1800 cm^−1^.

Vickers hardness was measured with a microhardness tester. The experimental loading time was 10 s, and the loading pressure was 1.96 N; each sample was tested 5–10 times to reduce experimental errors and improve the accuracy and reliability of the experiment. Archimedes’ buoyancy method was used to measure the density of the samples, and the molar volume was calculated according to Formulas (1) and (2) combined with the density. The dynamic method was used to measure the thermal expansion coefficient of the sample. The instrument used was an American Orton DIL2010STD thermal expansion instrument. The shaped and annealed glass samples were cut into strips with a length × width × height of 25.4 × 8 × 5 mm, the heating rate was 5 °C/min, and the heating temperature range was 25~500 °C.The shaped and annealed glass samples were cut into strip samples with a size of 25.4 × 8 × 5 mm, at a heating rate of 5 °C/min and a heating temperature range of 25~500 °C. The sample was cut into a size of 30 mm × 30 mm × 1 mm and optically polished for chemical stability testing. The glass slides were immersed in a 10 vol% hydrofluoric acid solution at room temperature for 30 min, and some glass plates were immersed in a solution of equal volumes of 1 mol/L NaOH and 1 mol/L KOH at 90 °C for 4 h. The chemical stability of glass is mainly expressed by the weight loss ratio (WLR) [22].

(1) $$\rho_{g}=\frac{M}{M-m}*\rho_{w}$$

where ρ_g_ is the density of the glass sample; ρ_w_ is the density of water at room temperature, and the unit of density is g/cm^3^; M is the mass of the glass in air (g); and m is the equilibrium reading of glass in water (g).

(2) $$V_{M=}\;\frac{\sum M_{i}x_{i}}{\rho_{g}}$$

where ρ is the glass density; x_i_ is the mole fraction of oxide component I; and M_i_ is the molecular weight.

(3) $$\mathrm{WLR}=\frac{M_{1}-M_{2}}{S}*100\%$$

where m_1_ is the mass of ultrasonic cleaning before etching; m_2_ is the mass of ultrasonic cleaning after etching; and s is the surface area of glass sample.

## 3. Results

### 3.1. Structural Analysis

#### 3.1.1. ^27^Al NMR Analysis

**Figure 1:**
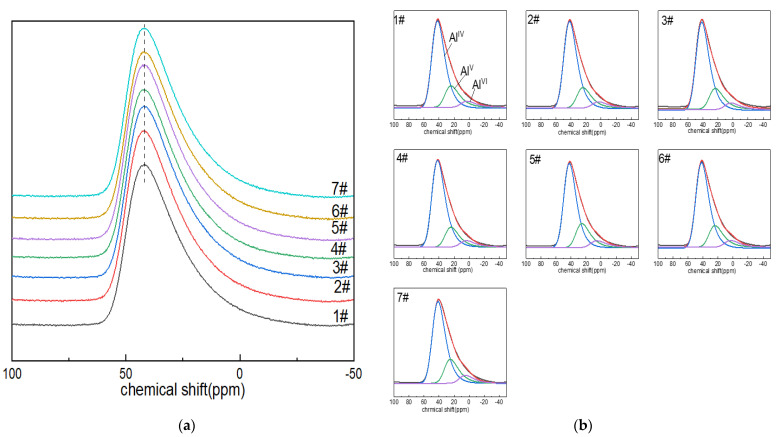
(**a**) ^27^Al MAS NMR images of Li_2_O−Al_2_O_3_−SiO_2_ glass with different B_2_O_3_ contents; (**b**) fitting diagram of ^27^Al MAS NMR peaks of Li_2_O−Al_2_O_3_−SiO_2_ glass with different B_2_O_3_ contents.

The ^27^Al NMR spectra of boron-doped lithium aluminum silicate glass are shown in Figure 1a. The ^27^Al MAS NMR peaks of the seven glass samples were all asymmetric composite peaks with a relatively broad distribution due to the quadrupole-broadening and localized-bonding environment related to compositional complexity, and the chemical shift in the resonance peak was ~42 ppm; the peak shapes were also roughly similar, indicating that the morphologies of the aluminum in the glass samples were relatively similar. With the increase in boron oxide content, the chemical shift in the peak shifted to high field, which may have been due to the fact that the oxygen in the Al–O bond carries low formal charge, and the amount of non-bridging oxygen in the Al–O network may be decreasing or the second element adjacent to it may change (B^III^ or Si is preferably to B^IV^ and Al^IV^) [41]. In addition, the spectral peak widened asymmetrically toward the low-chemical-shift direction, which may have been related to the appearance of the high coordination of aluminum ions and the change in quadrupole-coupling parameters.

In order to describe the coordination state of aluminum ions in the network structure and their respective contents, the reference and DM-fit software were used to fit and analyze the spectral peaks of the ^27^Al nuclear magnetic resonance of lithium aluminum silicate glass with the GIM model. GIM model fitting parameters included the following: the FWHM CS value was set to 14; the EM au value was set to −200; the d value was set to 5. The size of the computed spectra showed good rendering for 2048, but it was better with 8192, which was the preferred value. Step CSA, i.e., the step for sampling the isotropic CSA distribution in amorphous models, is typically 50, depending on the apodization factor. Step nuQ, i.e., the step for sampling the Gaussian distribution of quadrupolar interaction in amorphous or int2Q models, is typically 21, which can give decent results depending upon the final apodization factor. Sw mult, i.e., the sweep width multiplier, should be set to 4 in principle.

The chemical shift in the Al^IV^ peak in the network structure is about 45.8 ppm [42]. The peak shifts of Al^V^ and Al^VI^ are around 38–28 ppm and (–5)–15 ppm [43]. The GIM model fitting and calculation results of ^27^Al NMR spectra are shown in Figure 1b and Table 1. In the alumina network of boron-doped LAS glass, aluminum was always four-coordinated. The content of Al^V^ was only second to that of Al^IV^. It can be seen from Table 2 that with the increase in boron content, the contents of Al^IV^, Al^V^ and Al^VI^ in the aluminum–oxygen network changed little, and the spectral peak of Al^IV^ gradually moved toward the direction of low chemical shift.

#### 3.1.2. ^11^B NMR Analysis

**Figure 2:**
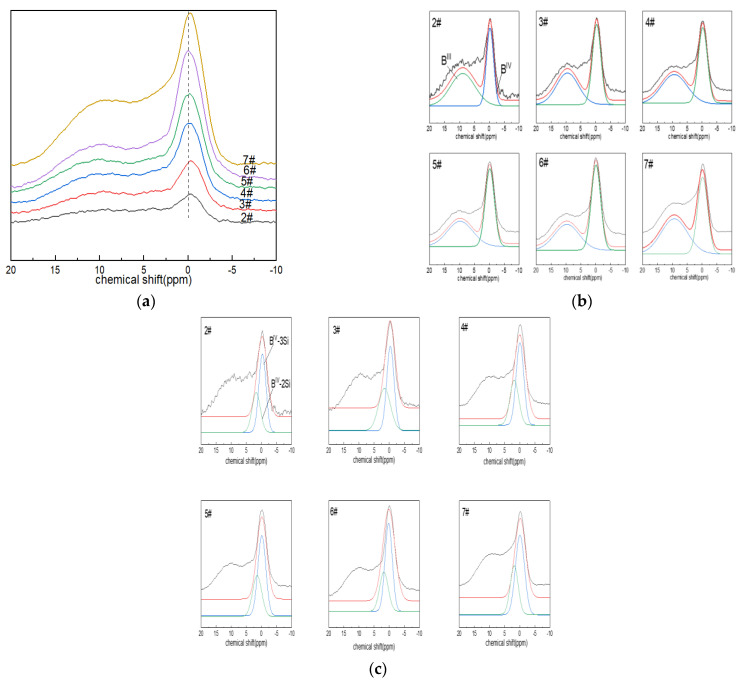
(**a**) ^11^B MAS NMR images of Li_2_O−Al_2_O_3_−SiO_2_ glass with different B_2_O_3_ contents; (**b**,**c**) fitting diagram of ^11^B MAS NMR peaks of Li_2_O−Al_2_O_3_−SiO_2_ glass with different B_2_O_3_ contents.

Figure 2a shows the NMR spectra of glass ^11^B. When a relatively weak magnetic field of 9.39 T was used, the NMR spectra contained two parts of the signal that could be resolved, which was consistent with the three-coordinated and four-coordinated boron atoms in the glass structure. It appeared that the symmetrical and relatively narrow Gaussian line shape in the ~0 ppm region was attributed to the [BO_4_] tetrahedron (i.e., B^IV^), while the complex-shaped, low-intensity asymmetric broad signal with chemical shifts of NMR peaks close to ~10 ppm corresponded to [BO_3_] units (B^III^). The chemical shifts of [BO_3_] and [BO_4_] associated with the two species changed little, so the properties of the entities did not appear to change significantly during the substitution. With the increase in boron oxide content, the absorption intensity of the boron-oxygen tetrahedral NMR spectral peak located at ~0 ppm increased gradually, that is, the content of B^IV^ increased.

The ^11^B NMR peaks were deconvolved with a Gaussian model to determine the components (B^III^ and B^IV^). The area percentages of B^IV^ and B^III^ were calculated, along with their respective contents (N_4_ and N_3_; N_4_ + N_3_ = 1). The connectivity of the silicate and borate networks could also be reflected by ^11^B NMR, and the fitting results are shown in Figure 2c. The connection of four-coordinated boron atoms with silicon atoms included: B^IV^-2Si (one four-coordinated boron atom linked to two silicon atoms via bridging oxygen), ~2.5 ppm; and B^IV^-3Si (one four-coordinated boron atom connected with three silicon atoms via bridging oxygen), ~0.5 ppm [43].

From the fitting results in Figure 2b,c and Table 3, it can be seen that the network structure of glass was dominated by B^IV^. The bonding states of free oxygen and boron atoms provided by alkaline earth metals or alkali metal oxides were related to their molar ratio, that is, Ψ = (O-Al_2_O_3_)/B_2_O_3_ (where O is the molar number of free oxygen); when Ψ ≥ 1, boron mainly existed in tetra-coordination. In the boron-doped glass system, when all Ψ > 1, the network structure was dominated by B^IV^. With the increase in the boron oxide content, N_4_ increased, and the area percentage of the B^IV^-3Si spectral peak at ~0.5 ppm was always more than the B^IV^-2Si spectral-peak area percentage at ~2.5 ppm.

#### 3.1.3. ^29^Si NMR Analysis

**Figure 3:**
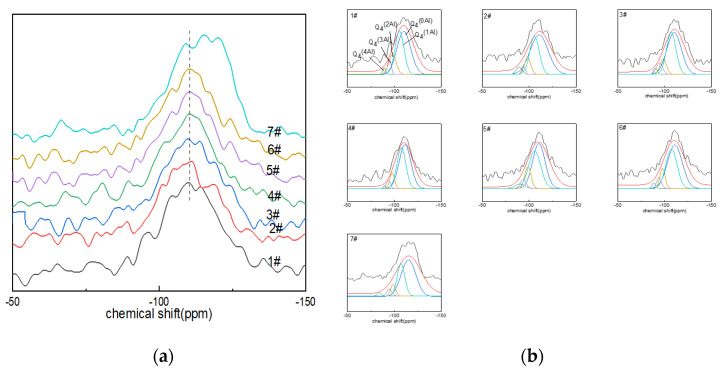
(**a**) ^29^Si MAS NMR of Li_2_O−Al_2_O_3_−SiO_2_ glass with different B_2_O_3_ contents; (**b**) fitting diagram of ^29^Si MAS NMR peaks of Li_2_O−Al_2_O_3_−SiO_2_ glass with different B_2_O_3_ contents.

The peaks of the ^29^Si NMR spectra ranged from −90 to ~−130 ppm (Figure 3a), centered on a broad resonance of −110 ppm. With the increase in boron oxide content, the spectral peaks tended to shift to the low field of ~−150 ppm, which was related to the existence of aluminum or boron atoms in the silicon–oxygen tetrahedron and the non-bridging oxygen number in the silicon–oxygen tetrahedron. The chemical shift in the spectral peaks of glass sample No. 7# was the most obvious. The presence of boron or aluminum resulted in a shift in the chemical shifts, and the substitution of [SiO_4_] with Al^IV^ or B^IV^ increased the chemical shift values by approximately 5 ppm [44]. Similarly, the presence of non-bridging oxygen atoms within the silicon coordination polyhedron caused the peaks to shift toward the high fields due to the high formal charge carried by oxygen [41].

The deconvolution of the ^29^Si NMR spectra could quantify the quantity of Q_n_^m(Al)^ in the network, where 0 ≤ m ≤ n ≤ 4, with n being the coordination number of silicon and m the aluminum atom connected with the silicon atom through the bridging-oxygen quantity. Q_n_^m(Al)^ was used to describe the connectivity of the silicon–oxygen network and aluminum–oxygen network. The connection between silicon and four-coordinated aluminum included: Q_4_^(0Al)^ connected with four silicon atoms (−102~−115 ppm); Q_4_^(4Al)^ connected with four aluminum atoms (−82~−90 ppm); Q_4_^(3Al)^ connected with three aluminum atoms (−85~−94 ppm); Q^4(2Al)^ connected with two aluminum atoms (−92~−98 ppm); Q_4_^(1Al)^ connected with an aluminum element (−96~−107 ppm) [45]. The Gaussian model fitting results of the ^29^Si NMR spectra of glass are shown in Figure 3b and Table 4.

From the fitting calculation results of the ^29^Si NMR spectra (Figure 3b), it can be seen that the main species in the glass network structure was Q_4_^(0Al)^, followed by Q_4_^(1Al)^; the area percentage of the two exceeded 60%, and Q_4_^(3Al)^ and Q_4_^(4Al)^ contents were low. Among them, the Q_4_^(0Al)^ content of glass samples Nos. 1#~6# increased with the increase in boron oxide content, while the content of Q_4_^(0Al)^ of glass sample No. 7# decreased compared with that of No. 6#. However, the area percentage of Q_4_^(0Al)^ eventually dominated.

#### 3.1.4. Raman Spectroscopy

**Figure 4:**
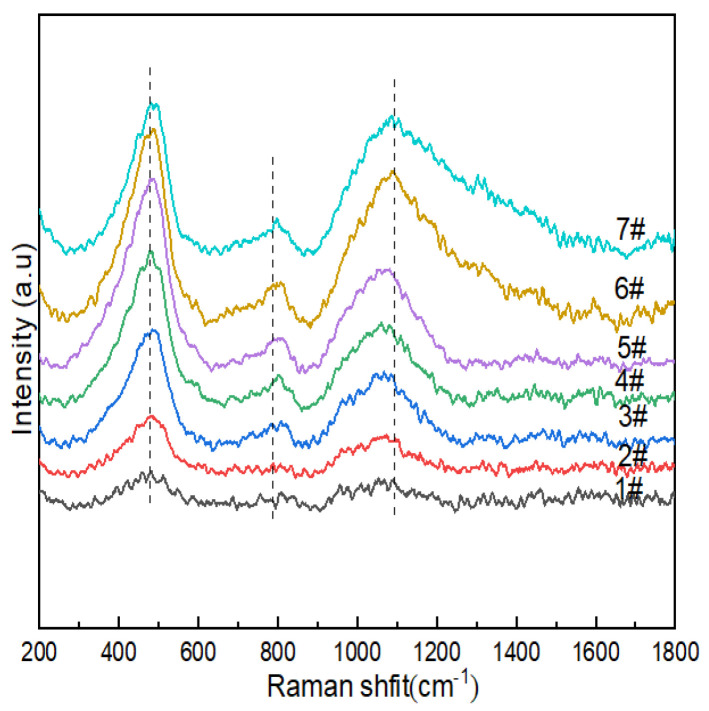
Raman spectra of Li_2_O−Al_2_O_3_−SiO_2_ system glass with different B_2_O_3_ contents.

Figure 4 shows the Raman spectra of lithium aluminosilicate glass. The relationships between the vibrations of the main functional groups in the spectra and the corresponding wave numbers are shown in Table 5. The dominant vibrations of the silicate network could be found in Si-O-Si within [SiO_4_] without non-bridging oxygen in the glass network structure in the lowest region between 200 cm^−1^ and 700 cm^−1^ corresponding to the T-O-T (where T is a Si atom) bending vibration. The bending vibrations and stretching vibrations of Q^n^ between 850 cm^−1^ and 1200 cm^−1^ corresponding to stretching were observed in the lowest region (n is the number of bridging oxygens in the silicon–oxygen tetrahedron).

The wave-number Raman spectra of the glass sample in the range of 200–1800 cm^−1^ are shown in Figure 4; there were characteristic peak intensity responses at 400–470 cm^−1^, 700–900 cm^−1^ and 900–1200cm^−1^. The vibrational peaks of 1#~7# were concentrated at ~480 cm^−1^, which indicated that more 5- and 4-membered rings formed in 2#, 3# and 4#. The peak at 470 cm^−1^ was attributed to the mixed tensile bending vibration mode of Si-O-Si, and with the increase in B_2_O_3_ (mol%), the peak intensities of 1#~6 # gradually increased, while the peak intensity of 7# decreased. Combined with ^29^Si NMR, it can be seen that the content of Q_4_^(0Al)^ decreased, and the connectivity of the silicate sub-network of glass changed. The vibrational peak at ~760cm^−1^ was the stretching vibration of the Si-O-Al linkages between [AlO_4_] and [SiO_4_] formed after Al^3+^ in the composition entered the network. The peak observed at ~770 cm^−1^ was attributed to the tetrahedral coordination of boron in glass due to the higher vibrational intensity of glass with high boron and low Na_2_O contents (e.g., 6#), while the opposite was true for glass with high Na_2_O and low B_2_O_3_ contents, which indicated that the modifier (such as sodium ions) functioned as a charge compensator, changing the neutral [BO_3_] unit into [BO_4_]^-^, thereby improving the aggregated state of the glass structure. It may have been formed by the breathing vibration of [BO_3_] of the three-membered ring at ~803cm^−1^. Combined with ^11^B NMR, it can be seen that its effect was relatively weak. In the range of 900-1200cm^−1^, we observed the Si-O stretching vibration in the silicon–oxygen tetrahedron, while the band at 920cm^−1^ could be attributed to the [BO_4_]^-^ containing structural unit, while the stretching vibration mode of the mixed Si-O-B linkages was also expected to function in this range [46]. In borosilicate glass, it is generally believed that for low-modified oxide additions (R < 0.5), triangular [BO_3_]^0^ converts to a [BO_4_]^-^ tetrahedron, and many NMR results are still interpreted to support the existence of [BO_4_]^-^ only being connected with the four silicate tetrahedra and not with the borate network. The boron content of the lithium–aluminosilicate glass system studied by the subject was low, and the band intensity related to borate was not as strong as the Si-O stretching vibration peak in the silicon–oxygen tetrahedron in the range of 900~1200 cm^−1^. With the increase in B_2_O_3_ (mol%), the peak intensity at 770 cm^−1^ gradually increased, which indicated that the content of [BO_4_] in glass increased, which was consistent with the calculation results of ^11^B NMR fitting. The band at 1080 cm^−1^ had a B-O stretching mode of tetrahedral borate units with a slight increase in peak intensity.

## 4. Discussion

### 4.1. Summary of the NMR and Raman Observations

The coordination state of aluminum ions in the glass network structure is related to the ratio of the alumina content in glass to the modifier oxide content, that is, Al_2_O_3_/M_2_O(MO) (where M is the modifier cation). When Al_2_O_3_:M_2_O(MO) < 1, a sufficient number of oxygen ions in the system convert aluminum ions into a tetrahedral coordination, and Al^3+^ acts as a network former [47]. In the boron-doped glass system, Al_2_O_3_:M_2_O(MO) < 1, and the number of oxygen ions was sufficient. Because Al^3+^ has a larger nuclear charge number than B^3+^, its ionic field is stronger, and the content of boron is smaller than that of aluminum, so it is more favorable for Al^3+^ to preferentially combine with free oxygen. Therefore, the incorporation of boron did not substantially affect the coordination of aluminum ions. The increase in the coordination of aluminum ions stemmed from the competition for short and strong oxygen bonds between modifier cations and aluminum atoms. Modifiers with smaller radii and higher charges have a stronger ability to compete for oxygen, which promotes the high coordination of aluminum ions [48].

When modifier oxide was added to glass to promote the formation of B^IV^, the oxygen ion (O^2−^) in the oxide destroyed the network connection, converted bridging oxygen (BO) to NBO, and changed the coordination of boron. The conversion relationship between B^IV^ and B^III^ is shown below.

B^IV^⇄B^III^ +NBO (4)

Modifier field strength, boron concentration, aluminum and total modifier content have important effects on the coordination of boron in a network structure [49]. At high temperatures, the reaction skews to the right. The effect of temperature on N_4_ depends on the NBO content, which, in turn, depends on the composition. Increasing the SiO_2_ content to a certain extent gives the possibility of reducing the B^IV^-O-B^IV^ linkages by dilution, and if the borate units in the network are less diluted by SiO_2_, the number of B^IV^-O-B^IV^ linkages increases [20]. Under the premise that the borate units in the network structure are less diluted by silica, network connections with relatively high formal charges, e.g., B^IV^-O-B^IV^ and Al^IV^-O-B^IV^, may increase. These linkages represent a charge concentration that is (−0.5) more negative than that of linkages such as B^III^-O-B^IV^ (−0.25), B^III^-O-B^III^(0) and ^[4]^Si-O-B^III^(0); the charge value is also expected to be stabilized to a certain extent by higher-field-strength-modified cations, which can better compensate for B^IV^-O-B^IV^ and the charge of Al^IV^-O-B^IV^ [49]. In B^IV^-O-B^IV^ and Al^IV^-O-B^IV^ linkages, BOs with higher negative charge concentrations compete with modifier cations for charge compensation more than the NBOs in the B^III^-O-B^IV^(−0.25) and B^III^-O-B^III^(0) linkages, which is more advantageous. Therefore, in glass with high modifier content, N_4_ is still high.

To test the interpretation of the ^29^Si NMR spectrum in terms of Q_n_^(mAl)^ composition, the main assumptions regarding the location of non-bridging oxygen, which is assumed to only be present in the silicon environment, and the alignment of aluminum–oxygen–aluminum bonds according to Lowenstein’s rule are first emphasized, with missing or negligible ratio. Furthermore, the properties of the Q_n_^(mAl)^ structural environment take into account the chemical shift ranges given by Engelhardt et al. [50] and are derived from extensive studies of crystalline mineral morphology [51].

The introduction of boron oxide resulted in a shift in the signal center of gravity towards the lower-chemical-shift direction and an increase in signal asymmetry with the emergence of separation towards the lower-chemical-shift direction. Therefore, this evolution of the ^29^Si NMR spectra may have reflected an increase in silicate network aggregation, especially due to the formation of Q_4_^(0Al)^ species, or a decrease in Si-O-Al linkages, which was to be expected here. With the addition of boron, the connectivity of Al in the tetra-coordinated silicon environment evolved, and the increase in Q_n_^(0Al)^ entities explained the shift in the spectrum to high fields. The content of Q_4_^(1Al)^ in the network structure was only second to that of Q_4_^(0Al)^, and the sum of the two exceeded 60%. The content of Q_n_^m(Al)^ (2 ≤ m ≤ 4) showed a decreasing trend as a whole with the increase in boron oxide content. The area percentage of Al^V^ was only second to that of Al^IV^, relatively speaking; the number of Al^IV^ entering the network framework decreased, which was beneficial to the formation of Q_4_^(0Al)^ [43]. The decrease in the content of Q_n_^m(Al)^ (2 ≤ m ≤ 4) meant that the connectivity between the pure silicon–oxygen network and the aluminum–oxygen network was weakened.

Raman spectroscopy showed increased interconnectivity of the borate and silicate sub-networks in the glass network, which was due to the fact that silicates consist of smaller rings and more mixed sub-networks of Si-O-B linkages, and the mixed Si-O-B linkages represented the coexistence of Si-O-B^III^ linkages and Si-O-B^IV^ linkages. From the perspective of the integrity of the Si-O-Si groups, B first enters the connecting bonds of the network in a layered [BO_3_] structure, which has a certain degree of negative impact on the integrity of the surrounding Si-O bonds. As a result, the bond fracture of the silica skeleton occurs, resulting in the destruction of the integrity of the silicon–oxygen–silicon group. From the uniformity of the structure distribution, when the layered conformation is inserted into the three-dimensional conformation and there is no obvious phase separation, the jamming phenomenon very easily occurs inside the network. This is also the reason why B_2_O_3_ can be introduced into some glass systems as a flux. Combined with the fitting results of NMR spectra, it could be inferred that the vibration peaks of [SiO_4_] with different bridging-oxygen numbers in the range of 800–1100 cm^−1^ were Q^n^, and Q^1^ mainly included Q_4_^(1Al)^; Q^2^ mainly included Q_4_^(2Al)^ and B^IV^-2Si; Q^3^ mainly included Q_4_^(3Al)^ and B^IV^-3Si; and Q4 mainly included Q_4_^(4Al)^.

### 4.2. Effect of Network Structure on Performance

#### 4.2.1. Thermal Expansion Analysis

**Figure 5:**
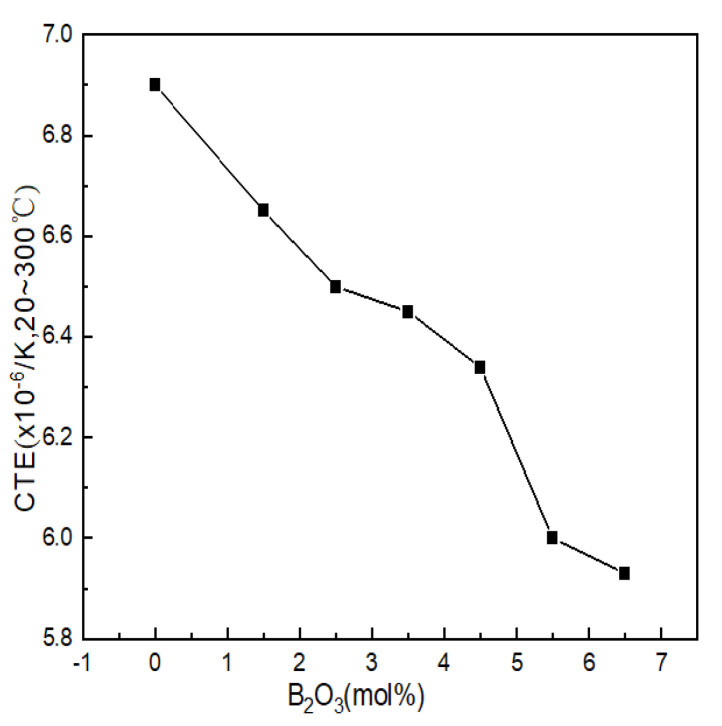
Curve of thermal expansion coefficients of Li_2_O−Al_2_O_3_−SiO_2_ system glass with different B_2_O_3_ contents.

The coefficient of thermal expansion reflects the ability of the glass structure to resist deformation when heated and is one of the ways to characterize the properties of glass. The thermal expansion curve of Li_2_O-Al_2_O_3_-SiO_2_ series glass with different B_2_O_3_ contents is shown in Figure 5. The thermal expansion coefficients of the seven groups of samples were 6.90 × 10^−6^/K, 6.65 × 10^−6^/K, 6.50 × 10^−6^/K, 6.45 × 10^−6^/K, 6.34 × 10^−6^/K, 6.00 × 10^−6^/K and 5.93 × 10^−6^/K.

B_2_O_3_ was arranged in the glass structure in the form of B^III^ and only relied on weak van der Waals forces to connect between the layered structures; the binding force that the particle vibration needed to overcome became smaller, resulting in an increase in the thermal expansion coefficient. During the appearance of highly coordinated boron (B^IV^) in glass, the bonding force of the structural connection was greatly strengthened within a certain range (mainly compared with the van der Waals force), and the layered structure of the glass was repaired and accumulated into a spatial grid, which directly led to the reduction in the thermal vibration amplitude of the particles. The smaller the distance between the particles was, the smaller the thermal expansion coefficient of the glass was; the influence of structural unit B^IV^ on the glass network structure was similar to that of the [AlO_4_] tetrahedron, and the broken network could also be reconnected. Therefore, B^IV^ had a positive effect on the thermal expansion of the glass. It can be seen from Figure 5 that after adding B_2_O_3_, the thermal expansion coefficient showed obvious regularity. The thermal expansion coefficients of glass samples 1#~7# decreased with the increase in boron oxide content, which was related to the network structure.

Combined with the GIM fitting calculation results of ^27^Al NMR, it can be seen that when the contents of B_2_O_3_ (mol%) were 2.5 and 6.5, the contents of Al^IV^ decreased when B_2_O_3_ (mol%) had values of 1.5 and 5.5, while the high-coordinated molecules entering the network were reduced. The amount of aluminum was relatively high, and the density of the network structure was relatively low. Therefore, when the content of B_2_O_3_(mol%) increased from 2.5 to 3.5 and from 5.5 to 6.5, the decreasing trend of the thermal expansion coefficient was relatively gentle.

#### 4.2.2. Microhardness

**Figure 6:**
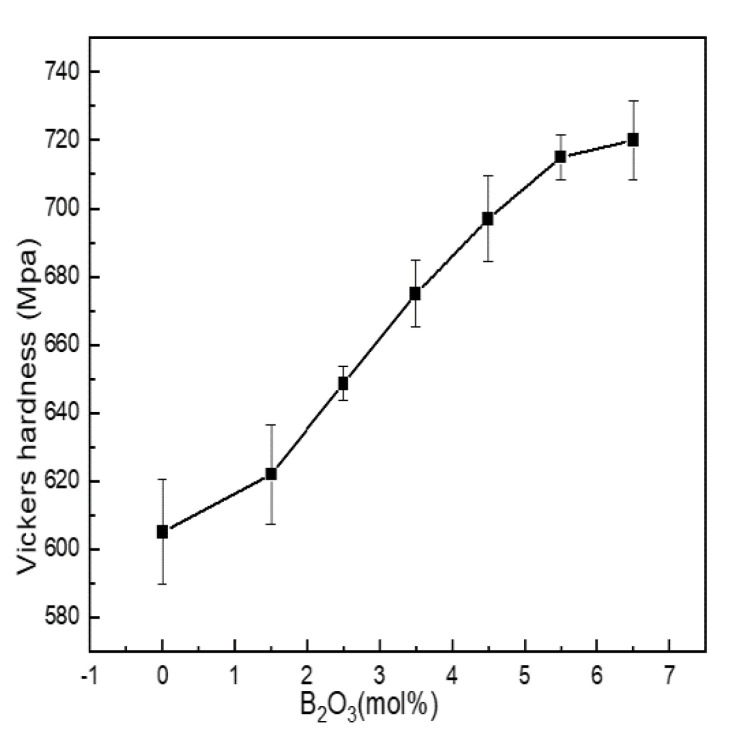
Change curve of mechanical properties of Li_2_O−Al_2_O_3_−SiO_2_ series glass with different B_2_O_3_ contents.

Microhardness is the most commonly used index to characterize the mechanical properties of materials. It is affected by the degree of aggregation of the network structure. Figure 6 shows the Vickers hardness of Li_2_O-Al_2_O_3_-SiO_2_ series glass with different B_2_O_3_ contents.

Vickers hardness was between 605 and 720 MPa. When the content of B_2_O_3_ (mol%) increased from 0 to 5.5, boron and aluminum mainly existed in four-coordinated forms, acting as network formers. N_4_ and Q_4_^(0Al)^ increased with the increase in boron content, and the network structure shrank, while the density and integrity of the structure were enhanced, and the ability to resist external force damage was enhanced.

When B_2_O_3_(mol%) = 6.5, the values of N_4_, Q_4_^(0Al)^ and Al^IV^ were all lower than when B_2_O_3_(mol%) = 5.5. The content of SiO_2_ in the glass exceeded 60 mol%, and the network modifiers containing N_3_, Al^V^ and Al^VI^ were not only connected with the silicon skeleton but also entered the glass cavity to improve the network density and the content of N^4^, while Q_4_^(0Al)^ and Al^IV^ always dominated. Therefore, the increased content of network modifier did not change the overall change trend of Vickers hardness but only slowed down its growth trend.

#### 4.2.3. Physical Properties

**Figure 7:**
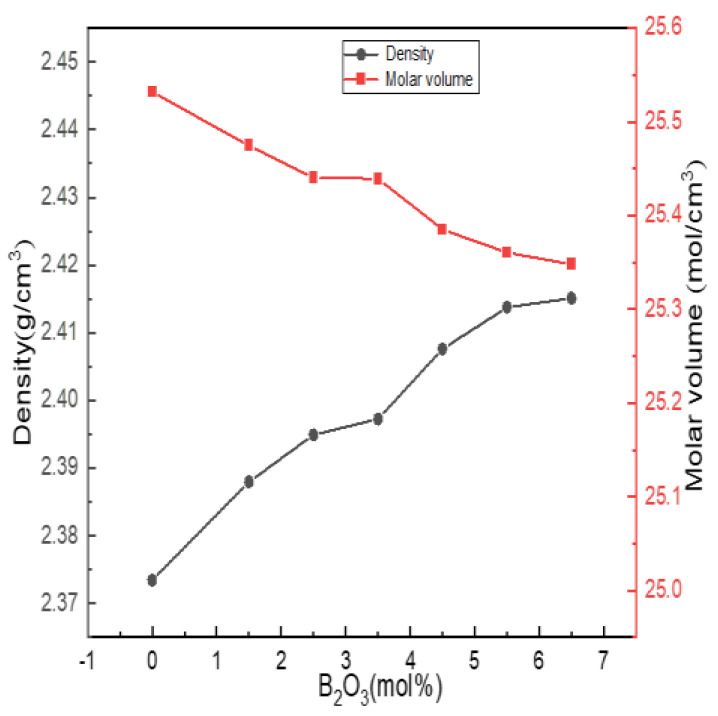
Change curves of physical properties of Li_2_O−Al_2_O_3_−SiO_2_ series glass with different B_2_O_3_ contents.

Density is the most basic performance parameter of an object. The density of glass is sensitive to changes in structure, and structural changes are basically reflected in changes in density. The molar volume of glass depends on the packing density of atoms in the glass network structure and the density and integrity of the glass network, which reflect the porosity of the glass structure. Figure 7 shows the experimentally obtained density and molar volume results.

It can be seen from Figure 7 that the density of the glass increased with the increase in the boron oxide content, and the molar volume gradually decreased with the increase in the boron oxide content. When the content of B_2_O_3_ (mol%) increased from 0 to 5.5, combined with the fitting calculation results of ^27^Al, ^11^B and ^29^Si NMR spectra, it can be seen that boron and aluminum atoms mainly existed in four-coordinated forms, acting as network formers; N_4_ and Q_4_^(0Al)^ increased, and the network structure shrank, while the structure density and integrity were strengthened.

When B_2_O_3_ (mol%) = 6.5, compared with B_2_O_3_ (mol%) = 6.5, the values of N_4_, Q_4_^(0Al)^ and Al^IV^ in the network structure decreased. However, the density change trend did not change, which was related to the way in which [BO_3_] was connected with the silica skeleton. The SiO_2_ content of the entire glass system exceeded 60 mol%. [BO_3_] and [BO_4_] in the high silicon glass system were mainly connected with the silica skeleton, and some [BO_3_] may have entered the glass cavity, such as the Al^V^ or Al^VI^ of the network modifier, causing the network structure to shrink. Therefore, the increasing trend of density became flat.

#### 4.2.4. Chemical Stability

**Figure 8:**
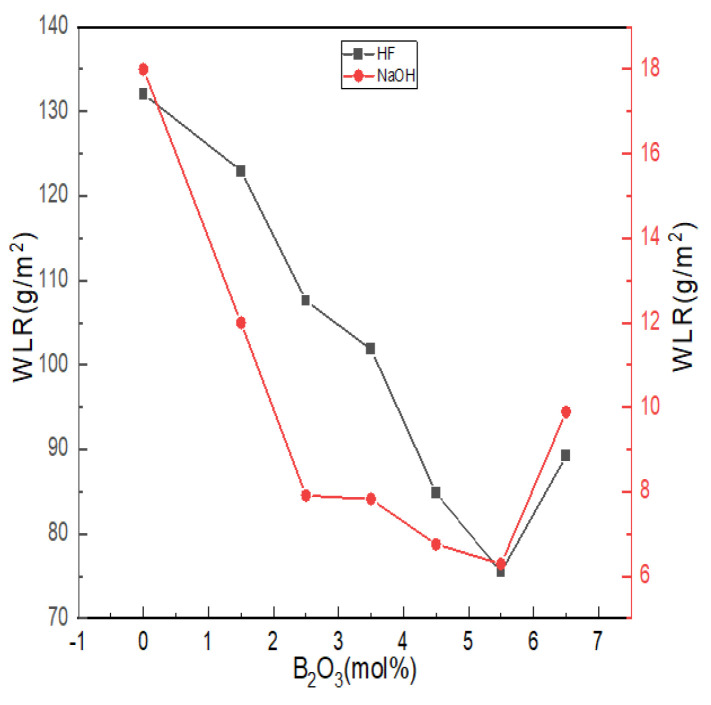
Chemical stability curves of Li_2_O−Al_2_O_3_−SiO_2_ series glass with different B_2_O_3_ contents.

The chemical stability of glass is affected by the composition, structure and other factors of glass. The erosion effect of acid on glass is to change, destroy or leach the network modifiers, such as alkaline earth metals or alkali metal oxides, in the glass structure; alkalis not only act on the network modifiers but also dissolve the silica skeleton of glass. Figure 8 shows the weight loss ratio of lithium aluminum silicate glass with different boron oxide content in strong acid and strong alkali solutions.

With the increase in boron oxide content, the weight loss ratio of glass in hydrofluoric acid and alkali solutions showed a trend that first decreased and then increased. The alkaline earth metal ions in the glass formed low-solubility silicates with the silicate ions generated after erosion and adhered to the glass surface to form a protective film, which can slow down further erosion, so the overall quality of glass lost in the alkaline solution was maintained at lower levels. The degree of damage of H^+^ and OH^-^ to the damaged network structure was related to the number of non-bridging oxygens in the network structure. According to the NMR spectra and the Raman test results, with the increase in boron content, the aluminum ion was dominated by tetra-coordination, while the values of N_4_ and Q_4_^(0Al)^ increased, and the number of bridging oxygens increased. The ternary network skeleton structures of [SiO_4_], [BO_4_] and [AlO_4_] tetrahedra were more compact, and the resistance of glass in hydrofluoric acid and alkali solutions increased. The reaction difficulty was increased, and the chemical stability was improved.

The acid resistance is not only affected by the structural integrity of a glass network but is also related to the cation species. Compared with Al^3+^, B^3+^ can enhance the acid resistance of glass within a certain range [22]. The corrosion of glass by HF mainly relies on the electrophilic corrosion of non-bridging oxygen by high concentrations of H^+^, which undergoes a substitution reaction with R^2+^ [52]. As more B^3+^ was introduced into bonding sites in the form of -Si-O-B^IV^, it was difficult for the electrophilic corrosion mechanism of HF in the form of ion exchange to occur in the glass, so the acid resistance of glass became better. Since the glass itself reacted with HF (Al_2_O_3_, SiO_2_, etc.), acid weight loss was generally at a high level.

When B_2_O_3_ (mol%) = 6.5, the weight loss rate of glass in hydrofluoric acid solution and alkali solution increased, and when B_2_O_3_ (mol%) = 5.5, the chemical stability of glass appeared to have an extreme value. When B_2_O_3_ (mol%) = 6.5, the contents of [AlO_6_] and [AlO_5_] in the glass were the highest. [AlO_6_] and [AlO_5_] generally cause the prominent Si-O bond to break around it. The unstable polar covalent bond requires more R^2+^ for charge neutralization and increases the sensitivity to acid or alkaline olutions due to its relatively relaxed structure that promotes the acceleration of ion transport.

## 5. Conclusions

In this paper, the effects of different contents of B_2_O_3_ (mol%) on the structure, thermal properties, chemical stability and mechanical properties of Li_2_O−Al_2_O_3_−SiO_2_ series glass were studied.

According to the fitting calculation results of the NMR spectra of ^27^Al, ^11^B and ^29^Si, it could be seen that the aluminum atoms in the network structure were dominated by four-coordinated Al^IV^ and that the content of Al^IV^ did not change with B_2_O_3_ (mol%); boron was mainly tetra-coordinated [BO_4_], and the N_4_ value increased with the increase in boron oxide content. The content of Q_4_^(0Al)^ in the silicon spectrum dominated and increased with the increase in B_2_O_3_ (mol%); at B_2_O_3_ (mol%) = 6.5, Q_4_^(0Al)^ decreased. The overall density of the glass network structure constructed by the borate network, the silicon–oxygen network and the aluminum–oxygen network increased with the increase in B_2_O_3_(mol%). Combined with the fitting results of the nuclear magnetic resonance spectra, it could be inferred that the types of [SiO_4_] vibration peaks in the range of 800–1100 cm^−1^ in the Raman spectra of different bridging-oxygen numbers were Q^n^, where Q^1^ mainly included Q_4_^(1Al)^; Q^2^ mainly included Q_4_^(2Al)^ and B^IV^-2Si; Q^3^ mainly included Q_4_^(3Al)^ and B^IV^-3Si; and Q4 mainly included Q_4_^(4Al)^.

The incorporation of boron oxide improved the degree of polymerization and connectivity of the glass network structure as a whole, and its corresponding mechanical properties and physical properties were also optimized. When B_2_O_3_ (mol%) = 5.5, the thermal expansion coefficient of glass was 6.00 × 10^−6^/K; the flexural strength and hardness were 180.665 Mpa and 715 Mpa, respectively; the density was 2.41g/cm^3^; and the acid resistance was improved by about 43%. On the whole, when B_2_O_3_ (mol%) = 5.5, the performance of glass was the best.

## Figures and Tables

**Table 1:** The quality contents of B_2_O_3_ in all samples (wt%). All data were only calculated for oxides.

Sample	2#	3#	4#	5#	6#	7#
Nominal ratio (%)	1.72	2.86	4	5.14	6.28	7.4
Proportion before melting (%)	1.93	3.20	4.48	5.76	7.03	8.29
Measured proportion after melting (%)	1.76	3.00	4.31	5.44	6.87	8.01
Volatility (%)	8.81	6.25	3.79	5.56	2.28	3.38

**Table 2:** Calculation results of GIM model fitting of ^27^Al NMR spectra.

Sample		Al^IV^	Al^V^	Al^VI^
1	Position (±0.2 ppm)	42.6	24.6	2.6
	Area (±3%)	73.0	20.2	6.8
2	Position (±0.2 ppm)	41.7	23.9	4.0
	Area (±3%)	74.1	19.0	6.9
3	Position (±0.2 ppm)	42.0	23.0	2.8
	Area (±3%)	74.8	18.7	6.5
4	Position (±0.2 ppm)	42.9	24.1	3.7
	Area (±3%)	75.3	18.3	6.3
5	Position (±0.2 ppm)	43.0	26.0	5.4
	Area (±3%)	75.6	16.8	7.6
6	Position (±0.2 ppm)	42.6	25.1	4.2
	Area (±3%)	73.4	20.2	6.4
7	Position (±0.2 ppm)	42.6	25.3	5.4
	Area (±3%)	69.0	23.5	7.5

**Table 3:** Calculation results of quadrupolar model fitting of ^11^B NMR spectra.

Sample		N_4_	N_3_	B^IV^-3Si	B^IV^-2Si
2	Position (±0.2 ppm)	−0.1	9.3	0.4	2.0
	Area (±3%)	50.2	49.8	65.0	35.0
3	Position (±0.2 ppm)	−0.1	9.4	0.3	2.5
	Area (±3%)	52.6	47.4	58.6	41.4
4	Position (±0.2 ppm)	−0.3	9.7	0.2	1.8
	Area (±3%)	54.4	45.6	63.2	36.8
5	Position (±0.2 ppm)	0.1	9.6	0.5	1.7
	Area (±3%)	55.8	44.2	62.5	37.5
6	Position (±0.2 ppm)	0	9.8	0.4	2.0
	Area (±3%)	59.9	40.1	65.9	34.1
7	Position (±0.2 ppm)	0	8.7	0.5	2.0
	Area (±3%)	58.0	42.0	60.9	39.1

**Table 4:** Calculation results of Gaussian model fitting of ^29^Si NMR spectra.

Sample		Q_4_^(0Al)^	Q_4_^(1Al)^	Q_4_^(2Al)^	Q_4_^(3Al)^	Q_4_^(4Al)^
1	Position (±0.2 ppm)	−108.9	−104.5	−97.6	−94.9	−88.3
	Area (±3%)	39.9	46.4	8.2	3.9	1.6
2	Position (±0.2 ppm)	−109.8	−104.8	−97.8	−92.7	−88.3
	Area (±3%)	44.9	36.7	10.7	4.3	3.4
3	Position (±0.2 ppm)	−110.6	−106.4	−97.8	−93.6	−90.8
	Area (±3%)	48.9	32.3	12.2	4.9	1.7
4	Position (±0.2 ppm)	−111.0	−107.7	−97.0	−93.4	−89.0
	Area (±3%)	50.5	33.3	8.3	6.0	1.9
5	Position (±0.2 ppm)	−109.8	−105.0	−98.9	−94.3	−89.5
	Area (±3%)	52.0	36.5	7.4	3.4	0.7
6	Position (±0.2 ppm)	−110.0	−107.2	−96.8	−94.1	−87.8
	Area (±3%)	53.7	32.0	8.3	4.3	1.7
7	Position (±0.2 ppm)	−114.4	−106.2	−97.8	−93.9	−83.5
	Area (±3%)	51.2	36.7	6.9	3.6	1.6

**Table 5:** Raman vibration groups of glass.

Peak Position/cm^−1^	Assignment
~480 cm^−1^	Si–O–Si bending vibration in [SiO_4_] without non-bridging oxygen in glass network structure
~760cm^−1^	Stretching vibration of Si–O–Al between [SiO_4_] and [AlO_4_]
~770 cm^−1^	Symmetrical breathing vibrations of the [BO_4_] group
~803cm^−1^	Boroxol ring breathing mode of the [BO_3_] group
	Stretching vibration of Si-O bonds in silicon–oxygen tetrahedron [SiO_4_]

## Data Availability

The datasets generated during and/or analyzed during the current study are available from the corresponding author upon reasonable request.
